# The emerging role of circular RNAs in drug resistance of non-small cell lung cancer

**DOI:** 10.3389/fonc.2022.1003230

**Published:** 2022-10-11

**Authors:** Tinghao Yan, Xinchen Tian, Fen Liu, Qingbin Liu, Qing Sheng, Jianlin Wu, Shulong Jiang

**Affiliations:** ^1^ Cheeloo College of Medicine, Shandong University, Jinan, China; ^2^ Clinical Medical Laboratory Center, Jining First People’s Hospital, Jining Medical University, Jining, China; ^3^ School of Architecture and Fine Art, Dalian University of Technology, Dalian, China; ^4^ School of Basic Medicine, Shandong University of Traditional Chinese Medicine, Jinan, China

**Keywords:** circRNAs, non-small cell lung cancer, drug resistance, miRNAs sponges, tumor microenvironment

## Abstract

Due to the characteristics of aggressiveness and high risk of postoperative recurrence, non-small cell lung cancer (NSCLC) is a serious hazard to human health, accounting for 85% of all lung cancer cases. Drug therapies, including chemotherapy, targeted therapy and immunotherapy, are effective treatments for NSCLC in clinics. However, most patients ultimately develop drug resistance, which is also the leading cause of treatment failure in cancer. To date, the mechanisms of drug resistance have yet to be fully elucidated, thus original strategies are developed to overcome this issue. Emerging studies have illustrated that circular RNAs (circRNAs) participate in the generation of therapeutic resistance in NSCLC. CircRNAs mediate the modulations of immune cells, cytokines, autophagy, ferroptosis and metabolism in the tumor microenvironment (TME), which play essential roles in the generation of drug resistance of NSCLC. More importantly, circRNAs function as miRNAs sponges to affect specific signaling pathways, directly leading to the generation of drug resistance. Consequently, this review highlights the mechanisms underlying the relationship between circRNAs and drug resistance in NSCLC. Additionally, several therapeutic drugs associated with circRNAs are summarized, aiming to provide references for circRNAs serving as potential therapeutic targets in overcoming drug resistance in NSCLC.

## Introduction

Lung cancer is a malignant tumor with high morbidity and mortality worldwide, with an evaluated 2.2 million new cancer cases and 1.8 million deaths in 2020 (1), and 85% of them are NSCLC (2). There have been significant advances in the treatment of NSCLC over the past several decades, particularly in targeting the mutations of epidermal growth factor receptor (EGFR) and anaplastic lymphoma kinase (ALK). In addition, immune checkpoint inhibitors, such as programmed death-1 (PD-1)/programmed death-ligand-1 (PD-L1) antibodies, have been used to treat driver gene-negative NSCLC (3). However, with the generation of drug resistance, the efficacy of chemotherapy and targeted therapy for NSCLC is greatly weakened. Hence, a better understanding of the drug resistance and identifying new therapeutic targets towards resistance are urgently needed.

Acquired drug resistance is one of the biggest challenges to clinical NSCLC treatment. The discovery of EGFR tyrosine kinase inhibitor (EGFR-TKI) effectively prolonged the remission and survival of patients with EGFR sensitive mutations in advanced NSCLC, mainly exon 19 deletions or the L858R point mutation in exon 21. However, almost all patients initially sensitive to the first or second generation EGFR-TKIs eventually developed drug resistance due to multiple molecular mechanisms. Normally, T790M mutation in exon 20 of EGFR gene is the most pervasive mechanism of acquired EGFR-TKI resistance (4). Consequently, it is essential to investigate effective solutions to manage drug resistance in NSCLC patients.

CircRNAs are derived from back splicing (5) with a closed-loop structure that emanates from the exosome. Since circRNAs have no 5’ or 3’ ends, they are resistant to RNA exonuclease-mediated degradation and thus are more stable (6). Accumulating studies showed that circRNAs are involved in multiple cellular biochemical processes of NSCLC, including proliferation (7), differentiation, metastasis, apoptosis and ferroptosis (8), demonstrating that circRNAs play a crucial role in NSCLC (9). Currently, a great number of literatures reported that the upregulated or downregulated expressions of circRNAs are closely associated with triggering NSCLC tumor cells to generate resistance to therapeutic drugs. In general, circRNAs mediate the development of drug resistance mainly through regulating miRNAs in NSCLC (10, 11). Furthermore, circRNAs can induce the occurrence of tumor drug resistance through multiple approaches, including inhibiting cancer cell apoptosis (12), accelerating drug excretion from cells (13), promoting DNA damage repair (14), maintaining the characteristics of tumor stem cells (15), and enhancing autophagy (16). Notably, in view of the relationship between circRNA and multidrug resistance, the unique back splicing of circRNA is a potential specific target for NSCLC, and precise regulation of circRNAs may play a therapeutic role in eliminating drug resistance. In this review, we summarize the recent findings of circRNAs in drug resistance of NSCLC, with the aim of providing references for overcoming drug resistance in NSCLC.

## The function of circular RNAs in NSCLC

**Figure 1:**
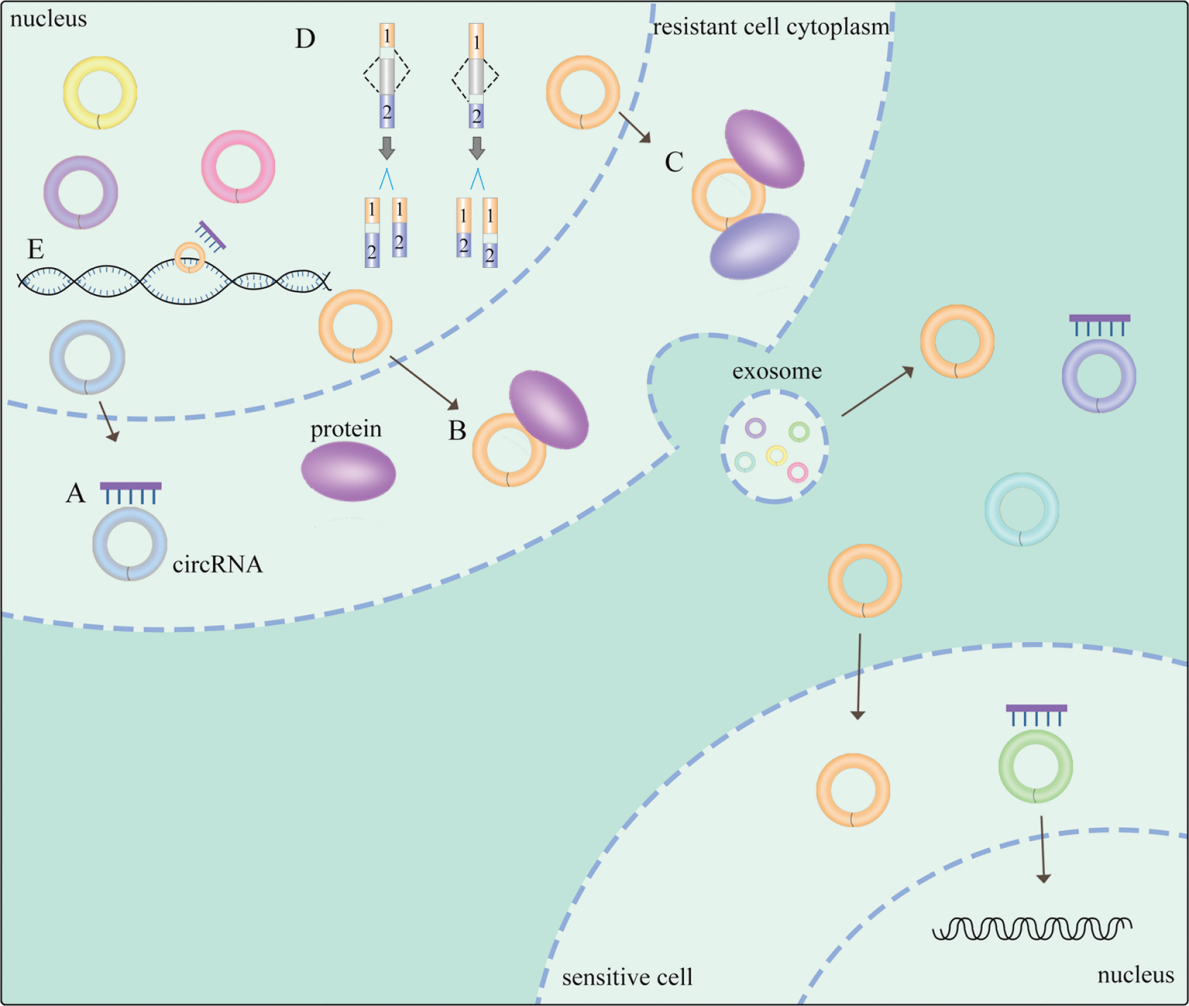
General mechanisms of circRNAs functions in NSCLC. **(A)** circRNAs act as miRNA sponges. **(B)** circRNA-protein interactions. **(C)** circRNAs function as protein scaffolding. **(D)** circRNAs modulate alternative splicing. **(E)** circRNAs regulate gene transcription. The meaning of "→" is "process".

Emerging evidence suggests that circRNAs employ several mechanisms to exert their biological functions in NSCLC (**Figure 1**). CircRNAs could function as sponges of miRNAs to form complexes to compete with endogenous RNAs (ceRNAs) (17), exerting a significant role of proliferation, apoptosis (18) and generating drug resistance (19) in NSCLC. Regulatory networks are formed when circRNAs attach to other molecules, constituting circRNA-DNA, circRNA-RNA, and circRNA-protein interactions (20). It is widely known that circRNAs with miRNA response elements (MREs) can operate as competitive molecules by binding to various miRNAs, reducing the ability of miRNAs to influence downstream mRNA expression (20). Secondly, circRNAs in the nucleus regulate alternative splicing (21, 22) and transcription (23). Moreover, circRNAs can serve as protein sponges or protein scaffolds in which circRNAs interact with some proteins (5), including specific RNA-binding proteins (RBPs) (24) to modulate proliferation and invasion in NSCLC (25). Thus, there exists a non-negligible relationship between circRNAs and NSCLC, while the potential mechanisms are still needed to be clearly investigated.

## The mechanisms of circRNAs mediating drug resistance of NSCLC

### Exosomes

Exosomes refer to a group of extracellular vesicles with sizes ranging between 30 and 150 nm (26). The exosomes are capable of transferring functional molecules, such as circRNAs, mRNAs (27), enzymes, and lipids (28), to neighboring or distant cells to regulate cellular activities. Thus, exosomes have a critical position in intercellular communication (29). The functional molecules of exosomes are associated with a variety of cancer-related activities, such as angiogenesis, metastasis, cell growth, survival and cancer stem cell renewal (30). It has also been shown that cancer cells secrete exosomes and cancer cell-derived exosomal circRNAs could serve as tumor markers (31). In human serum exosomes, it has been discovered over 1000 circRNAs, which are 6 times more than linear RNA. In addition, circRNAs derived from exosomes are found to promote resistance of NSCLC cells to chemotherapy, targeted therapy, and immunotherapy. Serum-derived exosomes express high levels of hsa_circ_0014235, which increases cisplatin(CDDP) chemoresistance in NSCLC cells (32). Yu et al. demonstrated that circ_0001658 induced TWIST1 expression through sponging miR-409-3p to promote gefitinib resistance in NSCLC (33). Also, Ma et al. demonstrated that hsa_circ_0002130 could contribute to osimertinib resistance in NSCLC through sponging miR-498 (34). Additionally, Chen et al. illustrated that cancer cell-derived exosomal circUSP7 could induce CD8+T cell dysfunction and anti-PD1 resistance by modulating the miR-934/SHP2 axis of NSCLC (11). Therefore, it is worthy to deeply understand the primary target molecules and signaling pathways of circRNAs that affect the acquisition of drug resistance, to establish a solid theoretical foundation for circRNA-based treatments.

### MiRNAs sponges

As post-transcriptional regulators of gene expression, miRNAs perform functions by pairing their seed region directly with messenger RNAs of protein-coding genes (35). Meanwhile, many circRNAs exhibit dominant biological functions by acting as miRNAs sponges (36). More specifically, circRNAs in the cytoplasm are identified to pair with complementary binding sites of miRNAs to regulate target gene expression (37). A growing number of studies have revealed that circRNA serving as a miRNA sponge is one of the major mechanisms of drug resistance in NSCLC. Zheng et al. found that the expression of circPVT1 was positively contributed to CDDP and pemetrexed chemotherapy resistance *via* modulating miR-145-5p/ABCC1 axis in lung adenocarcinoma (LUAD) (38). Zhang et al. suggested that circSOX13 was significantly overexpressed in NSCLC. The elevated circSOX13 was able to increase the malignant behavior and CDDP resistance of NSCLC *via* binding to miR-3194-3p (39). In addition, circ_PRMT5 was demonstrated to promote CDDP resistance by sponging miR‐4458, resulting in the overexpression of miR‐4458 targeted gene REV3L in NSCLC (40). Moreover, Zhou et al. found that enforced expression of PDPK1 could reverse the effects of knockdown of has_circ_0004015 on gefitinib sensitivity in NSCLC cells (41). Despite these findings, there still exist numerous circRNAs by binding miRNAs to control drug sensitivity of NSCLC. As a result, targeting the function of circRNA as a miRNA sponge could be an ideal therapeutic strategy to overcome drug resistance of NSCLC.

### Autophagy

Autophagy, a cellular “self-digestion” process, is a vital biological process involved in cellular survival. It has been demonstrated that the interaction between autophagy and TME significantly affects tumor progression (42). During long periods of tumor cells dormancy, autophagy can be induced by cancer therapeutic drugs and frequently contributes to cancer cell survival and the eventual outgrowth of tumors. On the one hand, autophagy is the recycling of degrading cellular metabolites for cellular survival (43). On the other hand, the proteins involved in various stages of autophagy regulate the apoptotic pathway (44). Accumulating evidence indicates that circRNAs influence drug resistance by the regulation of autophagy (45). For instance, circ_0085131 as a molecular sponge of mir-654-5p to overexpress autophagy-associated factor ATG7, leading to CDDP resistance of NSCLC (46). In addition, Zhong et al. found upregulation of circ_100565 regulated autophagy, proliferation and apoptosis, contributing to CDDP resistance of NSCLC (16). The underlying mechanism was that circ_100565 served as a sponge of miR-377-3p and overexpression of circ_100565 led to the increasing expression of miR-377-3p targeted gene ADAM28. Another study showed that circEHD2 governed the proliferation and glycolysis of NSCLC, but refrained autophagy and apoptosis through binding to miR-3186-3p targeting FOXK1, curbing the malignant phenotype of NSCLC (47). Whether circEHD2 can mediate drug resistance by affecting autophagy deserves further exploration.

### Ferroptosis

Ferroptosis is a newly identified form of cell death mediated by iron metabolism and oxidative stress (48). Currently, ferroptosis has been identified to associate with the development and therapeutic resistance of NSCLC (49–52). Thus targeting ferroptosis can be a relatively novel therapeutic approach for NSCLC treatment. One recent study showed that depletion of USP35, a member of deubiquitinases family, can boost ferroptotic cell death and enhance the sensitivity of cisplatin and paclitaxel chemotherapy in lung cancer cells (53). Another study reported that inhibiting glutathione peroxidase 4 could surmount resistance to lapatinib by increasing ferroptosis in NSCLC patients (54). Notably, numerous researches about ferroptosis have focused on the function associated with circRNAs. Li et al. recently discovered that circFOXP1 promoted malignant development of lung cancer by suppressing ferroptosis (55). Wang et al. revealed that circDTL served as an oncogene by regulating apoptosis and ferroptosis through the miR-1287-5p/GPX4 axis during the development of NSCLC (8). Therefore, it also deserves to be further investigated whether circRNAs could mediate therapeutic resistance of NSCLC *via* regulating ferroptosis.

### Metabolism

Emerging studies have shown that the metabolic reprogramming of TME has far-reaching ramifications for anticancer treatment resistance (56). In order to increase ATP production, malignancies reprogramme metabolism of tumor cells to oxidative phosphorylation (OXPHOS) in response to pharmacological treatment. Cancer-associated fibroblasts (CAFs) may participate in cancer cell-autonomous pathways to generate therapy resistance by promoting OXPHOS behavior and providing energy-rich foods in specific settings (57, 58). In addition, hypoxia-inducible factor 1a (HIF-1a), a transcription factor, regulates a large number of gene products involved in energy metabolism and glycolysis contributed to anticancer drug resistance (59). Currently, the relationship between circRNA and metabolism in NSCLC has gained a great deal of attention. Xu et al. discovered that knockdown of circAKT3 clearly decreased HIF-1a-dependent glycolysis and improved lung cancer cells sensitivity to CDDP by targeting the miR-516b-5p/STAT3 axis (60). Shi et al. reported that circ_0008928 silencing could enhance CDDP sensitivity and inhibit glycolysis metabolism by downregulating miR-488/HK2 Axis in CDDP-resistant NSCLC (61). Evidently, it is worthwhile to further explore the relationship between circRNAs and altered metabolism, thus helping to address the problem of drug insensitivity in NSCLC cells (The potential mechanisms of circRNAs in NSCLC are shown in **Table 1**).

**Table 1:** The potential mechanisms of circRNAs in NSCLC.

Mechanisms	CircRNAs	Targets	Effects	References
exosomes	hsa_circ_0014235	miR-520a-5p/CDK4 axis	contributes cisplatin-resistance in NSCLC	(32)
	hsa_circ_0002130	miR-498	contributes osimertinib-resistance in NSCLC	(34)
	circUSP7	miR-934/SHP2 axis	contributes anti-PD1 resistance in NSCLC	(11)
miRNAs spognes (ceRNAs)	CircPVT1	miR-145-5p/TAGLN2	contributes cisplatin and pemetrexed resistance in NSCLC	(38)
	circSOX13	miR-3194-3p/MAPREl axis	contributes cisplatin resistance in NSCLC	(39)
	Circ_PRMT5	miR‐4458/REV3L axis	contributes cisplatin resistance in NSCLC	(40)
	hsa circ_0004015	miR-1183/PDPK1 axis	contributes gefitinib resistance in NSCLC	(41)
autophagy	circ_0085131	autophagy-associated factor ATG7	contributes cisplatin resistance in NSCLC	(46)
	circEHD2	miR-3186- 3p/FOXK1 axis	expedites autophagy and apoptosis of NSCLC	(47)
	circ_100565	miR-337-3p/ADAM28 axis	contributes to cisplatin resistance of NSCLC cells	(16)
	circHIPK3	miR124-3p-STAT3-PRKAA/AMPKα axis	modulates autophagy	(62)
ferroptosis	circFOXP1		suppresses lung adenocarcinoma cell survival	(55)
	circDTL	miR-1287-5p/GPX4 axis	regulates apoptosis and ferroptosis in NSCLC	(8)
	circ_101093		ferroptosis desensitization in lung adenocarcinoma	(63)
metabolism	circAKT3	miR-516b-5p/STAT3 axis	regulates sensitivity to cisplatin and glycolysis in NSCLC	(60)
	circ_0008928	miR-488/HK2 Axis	regulates cisplatin sensitivity, and glycolysis metabolism in NSCLC	(61)
	circPTK2	miR-942/TRIM16 axis	overexpression of circPTK2 reduced cisplatin resistance and suppressed glycolysis of DP in NSCLC	(64)

### RNA-binding proteins

**Figure 2:**
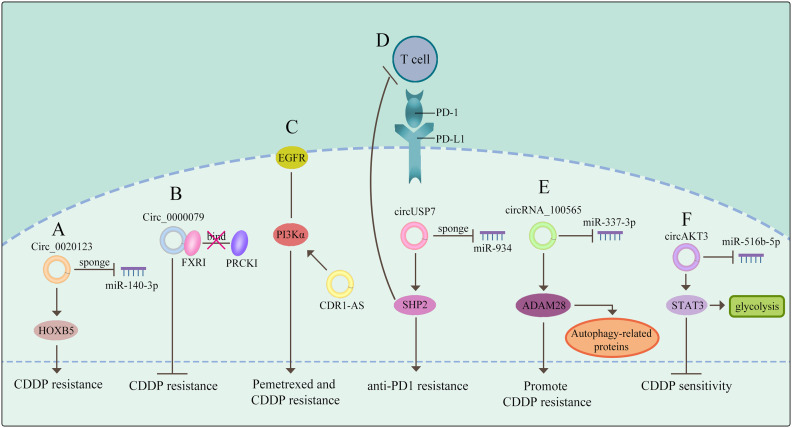
CircRNAs mediate drug resistance of NSCLC **(A)** circRNAs function as miRNA sponges to mediate drug resistance. **(B)** circRNAs govern drug sensitivity as the sponges of RBPs. **(C)** circRNAs mediate drug resistance through a signaling pathway. **(D)** circRNAs control drug resistance by inhibiting T cells. **(E)** circRNAs regulate autophagy derived proteins to mediate drug resistance. **(F)** circRNAs control drug resistance by curbing glycolysis. The meaning of "→" is "promote", and "T" is "inhibit".

Expect for functioning as miRNAs sponges to generate drug resistance, several literatures reported that circRNAs could modulate drug sensitivity as the sponges of RNA-binding proteins (RBPs). Chen et al. demonstrated that circ_0000079 could decline tumor cell invasion and CDDP resistance in NSCLC by interfering the formation of the FXR1/PRCKI complex (65). Another study found that circ_GRHPR interacted with the RBP PCBP2 could boost NSCLC cell proliferation and invasion, while it is still needed to identify whether circ_GRHPR could induce drug resistance of NSCLC (25). Consequently, a better understanding of the interaction between circRNAs and RBPs is beneficial to discover a novel therapeutic target for conquering drug resistance. (The potential mechanisms of circRNAs mediating drug resistance of NSCLC are shown in **Figure 2**)

## Signaling pathways associated with circRNAs in NSCLC

### MAPK Signaling Pathway

The members of mitogen-activated protein kinase (MAPK) family integrate signals that impact proliferation (66), differentiation, survival (67), migration, and tumorigenesis in a cell context and cell type specific manner (68). Of note, the transcriptional regulator inhibitor of differentiation could activate the p38MAPK pathway to promote chemoresistance by increasing stemness in cancer cell populations (69, 70). Recently, several circRNAs have been illustrated to be dysregulated in NSCLC and to govern the course of carcinogenesis *via* regulating the MAPK signaling pathway. Zhang et al. reported that has_circRNA_101237, was frequently overexpressed in NCSLC and knockdown of circRNA_101237 reduced cell proliferation, migration and invasion. Mechanistically, circRNA_101237 functions as a sponge of miR-490-3p targeting MAPK1 (71). Wang et al. demonstrated that circ-ZKSCAN1 can sponge carcinogenic miR-330-5p to elevate the level of FAM83A, leading to the suppression of MAPK signaling pathway, thus facilitating NSCLC progression (72). However, further investigation is needed to completely understand the role of circRNAs mediating drug resistance in NSCLC through the MAPK pathway.

### Wnt Signaling Pathway

Aberrant modifications of Wnt/β-catenin (73) are common, while the mutations of β-catenin (74) and APC (75) are rear in NSCLC. The activation of Wnt has been shown to promote drug resistance in NSCLC (76–78). Accumulating studies demonstrated that circRNAs could promote NSCLC development through the Wnt pathway activation. As proof, circ _0067934 is highly expressed in NSCLC and promotes tumor progression. In contrast, depletion of circ_0067934 hinders cell proliferation, migration, invasion and EMT and promoted apoptosis in NSCLC *via* inhibition of the Wnt/β-catenin pathway (79). Li et al. demonstrated that circCCT3 functions as a sponge of miR-107 to enhance invasion and EMT of NSCLC *via* regulating Wnt pathway and FGF7 (80). In addition, circ_PRKDC (81) activate Wnt pathway to induce 5-fluorouracil in colorectal cancer. Therefore, it is evident that circRNAs have a non-negligible part in drug resistance by regulating the Wnt signaling pathway. However, how circRNAs can cause drug resistance *via* Wnt signaling pathway in NSCLC needs to be further investigated.

### PI3K pathway

Phosphatidylinositol-3 kinases (PI3Ks), consist of a lipid kinase family characterized through generating the second messenger phosphatidylinositol-3,4,5-trisphosphate (PI-3,4,5-P3) (82). Subsequently, AKT is activated after interacting with these phospholipids, resulting in cell survival (83), cell cycle progression (84), and cellular proliferation (85). The components of the PI3K/AKT signaling pathway are commonly changed during cancer development. It has been shown that aberrant activation of the PI3K/AKT pathway was frequently involved in drug resistance (86). Amplification of MET could activate PI3K, leading to the development of TKIs resistance in lung cancer (87). Recent studies have revealed that aberrant expression of circRNAs affected the components of the PI3K signaling pathway to induce chemoresistance in NSCLC. For example, circ_CDR1-AS contributes to resistance to pemetrexed and CDDP through activating the EGFR/PI3K pathway in LUAD (88). Additionally, circ_0017639 (89) and circ_0008594 (90) both can facilitate the progression of NSCLC by PI3K signaling pathway. Therefore, circRNAs are potential targets for intervening in drug resistance problems, with circRNAs playing a significant role in the modulation of the PI3K/AKT pathway.

### STAT3 signaling pathway

**Figure 3:**
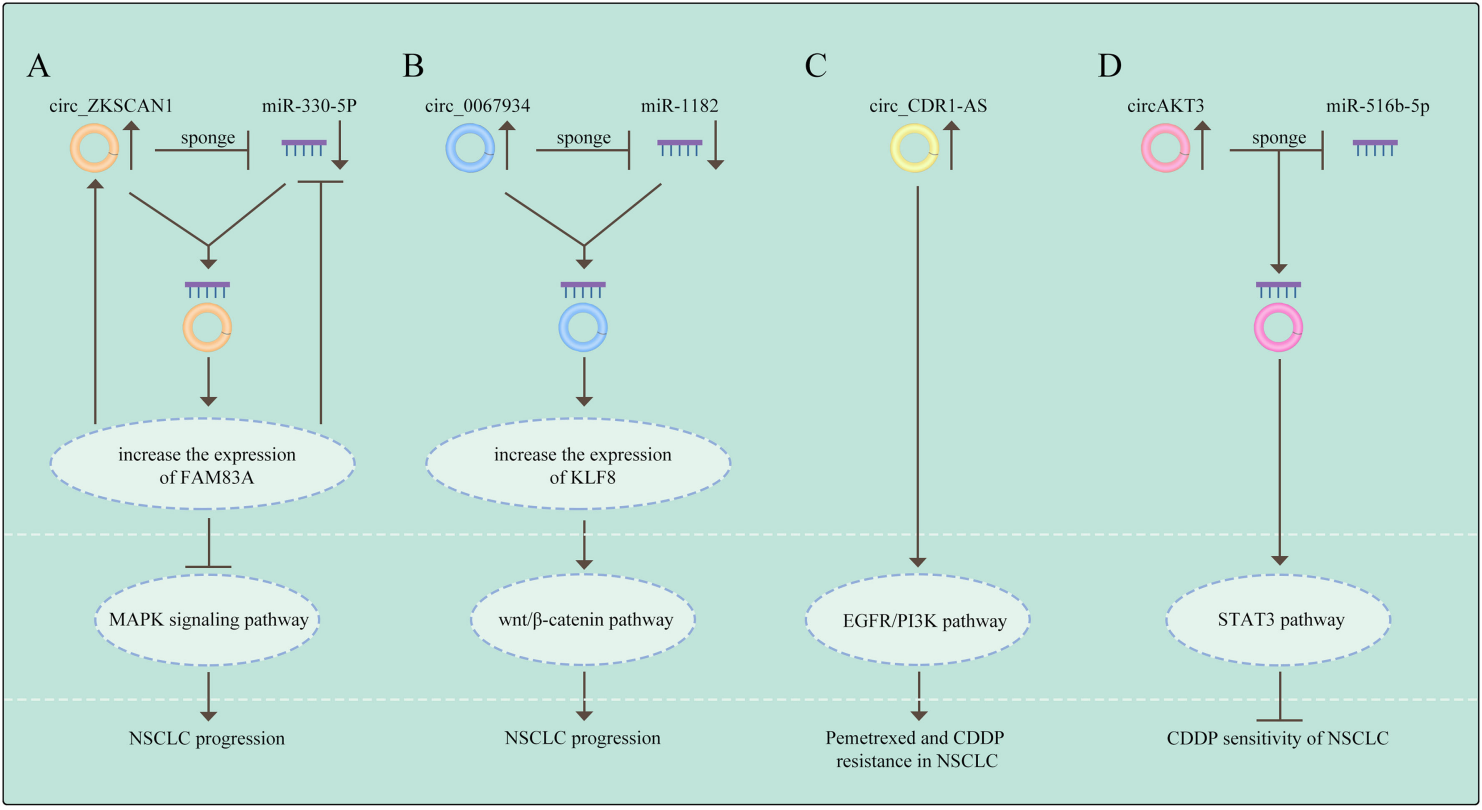
The relationship between signaling pathways and circRNAs in NSCLC **(A)** Circ_ZKSCAN1 sponges miR-330-5p to increase the expression of FAM83A, resulting in the inhibition of MAPK signaling pathway, thus promoting the progress of NSCLC. **(B)** Circ_0067934 promotes NSCLC development by regulating miR-1182/KLF8 axis and activating Wnt/β-catenin pathway. **(C)** CircRNA CDR1-AS contributes to pemetrexe and cisplatin resistance *via* the EGFR/PI3K signaling pathway. **(D)** CircAKT3 regulates cisplatin resistance of NSCLC *via* modulating STAT3 by sponging miR-516b-5p. The meaning of "→" is "promote", and "T" is "inhibit".

It has been indicated that pharmacological inhibition of the oncogene addiction pathways was related to feedback activation of the cell survival protein STAT3 and could therefore reduce the efficacy of drug therapy in NSCLC (91). So far, circRNAs also promote drug resistance *via* activating the STAT3 signaling. Dong et al. found that the expression of circ_0076305 was positively linked with STAT3 expression in NSCLC tissues and circ_0076305 could induce STAT3 expression through sponging miR-296-5p, thus leading to CDDP resistance in NSCLC (92). These results suggest that the STAT3 pathway is deeply involved in circRNAs-mediated drug resistance in NSCLC. (The relationship between signaling pathways and circRNAs in NSCLC are shown in **Figure 3**).

## Specific drug resistance associated with circRNAs in NSCLC

### Resistance to paclitaxel

Paclitaxel (PTX) is the first member of the taxane family to be employed in cancer treatment; taxanes cause cellular death by halting mitosis through the regulation of microtubule stability (93). It imposes the anti-tumor effects through interrupting the dynamics of microtubules, thereby leading to the mitotic block and cell death (94). PTX has become a widely treatment option for NSCLC patients, followed by the generation of PTX resistance in NSCLC. Guo et al. demonstrated that circ_0011292 promoted PTX resistance in NSCLC *via* modulating the miR-379-5p/TRIM65 axis, indicating that knocking down circ_0011292 might be a feasible therapeutic option for PTX resistance in NSCLC (95). Another study reported that the expression of circ_ZFR was elevated in PTX-resistant NSCLC, while knockdown of circ_ZFR was able to reverse PTX resistance by downregulation of KPNA4 *via* sponging miR-195-5p (96). Similarly, Xu et al. demonstrated that overexpression of hsa circ_0002874 induced PTX resistance by functioning as a sponge of miR1273f, suggesting that overexpression of circ_0002874 or ectopic expression of miR1273f could weaken PTX sensitivity in A549 cells (97). This study also revealed that hsa_circ_0002874 could be a potential PTX resistant biomarker in NSCLC (97). In addition, Li et al. showed that circ_0002483 overexpression increased the PTX sensitivity of NSCLC cells *via* sponging miR-182-5p, leading to the upregulation of miR-182-5p targeted genes GRB2, FOXO1, and FOXO3 (98). Collectively, these literatures demonstrate the prominence of circRNAs as miRNAs sponges in NSCLC PTX resistance, pointing to the possible options for NSCLC patients with PTX resistance.

### Resistance to docetaxel

Docetaxel (DTX), a highly efficient anticancer medication, is a standard therapy for NSCLC (99). DTX is beneficial in preventing the malignant progression of NSCLC and prolonging the survival of NSCLC patients (100, 101). Furthermore, evidence has clarified the relationship between dysregulation of circRNAs and DTX resistance in NSCLC. Du et al. showed that high level of circ_0014130 contributed to DTX resistance by regulating the miR-545-3p-YAP1 axis, and knockdown of circ_0014130 could reverse the chemoresistance in NSCLC cells (102). Another report found that circ_0003998 inhibited apoptosis and DTX sensitivity in DTX-resistant NSCLC by modulating the miR-136-5p/CORO1C axis. On the contrary, depletion of circ_0003998 rendered the resistant cells to regain the sensitivity of DTX (103). These investigations illustrated that DTX resistance have close associations with circRNAs in NSCLC, implying that it might be promising to identify circRNAs as the potential therapeutic targets for overcoming PTX and DTX resistance.

### Resistance to cisplatin

CDDP is widely used for the treatment of NSCLC as a common chemotherapeutic drug, however, a great number of studies have reported that circRNAs could hinder the clinical utility of platinum-based chemotherapy through diverse mechanisms (104). For instance, has_circRNA_103809 was overexpressed in CDDP-resistant NSCLC cells, sponging miR-337-3p to upregulate miR-337-3p targeted gene GOT1, and depletion of has_circRNA_103809 re-sensitized the NSCLC cells to CDDP (105). Chen et al. discovered that circ-CUL2 and RB1CC1 were downregulated, whereas miR-888-5p was upregulated in NSCLC cell lines. Besides, the upregualtion of circ_CUL2 inhibited A549/DDP cell growth and repressed CDDP resistance through sponging miR-888-5p/RB1CC1 axis (19). What’s more, circ_100565 sponging miR-377-3p was able to increase ADAM28 expression, leading to NSCLC cells resistance to CDDP. Circ_100565 was overexpressed in CDDP-resistant NSCLC and knockdown of circ_100565 could overrode the resistance (16). In addition, Zhang et al. identified that circSOX13 enhanced MAPRE1 expression by competitively binding miR-3194-3p, resulting in CDDP resistance in NSCLC cells (39). Similarly, circ_103762 expression was elevated following CDDP treatment in NSCLC patients. Overexpression of circ_103762 induced CDDP resistance and increased MDR expression by suppressing DNA damage inducible transcript 3 (CHOP) (106). Furthermore, Chang et al. found that circ_0017639 silencing inhibited tumor growth and enhanced CDDP sensitivity *in vivo*. Meanwhile, circ_0017639 also promoted apoptosis and suppressed proliferation, invasion, and migration of CDDP-resistant NSCLC cells *via* miR-1296-5p/SIX1 axis *in vitro* (107). These results indicate that circRNAs are supposed to be the functional biomarkers and novel therapeutic targets for NSCLC.

### Resistance to pemetrexed

Pemetrexed (PTX) has been a prominent focus in anticancer therapy research (108). In fact, pemetrexed and CDDP combination chemotherapy is frequently employed in the treatment of LUAD (109). Recently, Zheng et al. reported that elevated circ_PVT1 expression is linked to CDDP and pemetrexed insensitivity in NSCLC patients, and circ_PVT1 contributes to CDDP and pemetrexed resistance by miR-145-5p/ABCC1 axis (38). Mao et al. indicated that CDR1 Antisense RNA (CDR1-AS), an overexpressed circRNA in many tumors, promoted PTX and CDDP chemoresistance *via* regulating EGFR/PI3K signaling pathway in LUAD. Knockdown of circRNA-CDR1-AS could restore PTX and CDDP sensitivity in chemo-resistant LUAD cells; however, this effect was negated by the activation of EGFR/PI3K pathway (88). In view that pemetrexed resistance keeps constantly occurring in the chemotherapy of NSCLC, more circRNAs contributing to pemetrexed resistance would be uncovered in future.

### Resistance to gemcitabine

Gemcitabine is a first-line treatment option with significant clinical effects in NSCLC. Meanwhile, the combination of gemcitabine and CDDP exhibited a synergistic anti-tumor activity in NSCLC patients. Lu et al. found that circPVT1 expression was reduced after the combined therapy of CDDP and gemcitabine. Meanwhile, circ_PVT1 expression was higher in the chemotherapy-resistant group than the chemotherapy-sensitive group, indicating that circ_PVT1 expression is linked to chemotherapy resistance (110). To date, several studies have shown that circRNAs caused gemcitabine resistance in pancreatic cancer (PC). For example, circ_FARP1 operates as a ceRNA *via* sponging miR-660-3p to elevate LIF expression, ultimately activating the STAT3 signaling pathway and causing gemcitabine resistance in PC patients (111). Additionally, Yu et al. manifested that knockdown of circ_0092367 caused aggressive EMT features and gemcitabine resistance by regulating the miR-1206/ESRP1 axis in PC cells (112). Although most of the current literatures focus on the relationship between gemcitabine resistance and circRNAs in PC, gemcitabine resistance in NSCLC is also worthy of further investigation.

### Resistance to gefitinib, erlotinib, and osimertinib

Some cancer patients with specific genomic aberrations have benefited from targeted therapies (113). EGFR-TKIs (Gefitinib, Erlotinib, and Osimertinib) are the most common treatments for NSCLC with EGFR mutation (114). However, a majority of patients eventually develop resistance towards these therapies. Recent several studies have shown that EGFR-TKIs resistance may be associated with tumor-derived exosomal cirRNAs. For instance, Lu et al. reported that circ_RACGAP1 induced gefitinib resistance in NSCLC *via* miR-144/CDKL1 signaling cascade. Depletion of circ_RACGAP1 dramatically inhibited the cell cycle progression and reversed gefitinib resistance (115). Moreover, circ_0014235 acts as a sponge of miR-146b-5p to upregulate miR-146b-5p targeted gene YAP, leading to the increase of PD-L1 expression and immune escape, thereby promoting gefitinib resistance in NSCLC (116). In addition, Sheng et al. illustrated that overexpression of circ_SETD3 triggered gefitinib resistance by sponging miR-873-5p, whereas depletion of circ_SETD3 improved NSCLC cell sensitivity to gefitinib (117). Joseph et al. discovered that EGFR-TKI resistance was positively linked with the expression of circ_CCDC66, which was upregulated through FAK and c-Met but downregulated through nAchR7α (118).

Regarding the functions of circRNAs in the resistance of osimertinib, a third-generation EGFR-TKI, Liu et al. demonstrated that has_circ_0005576 promoted osimertinib resistance by regulating miR-512-5p/IGF1R axis in LUAD cells (119). Another report clearly showed that has_circ_0002130 expression was considerably increased in osimertinib-resistant NSCLC cells and serum exosomes from osimertinib-resistant NSCLC patients, suggesting circ_0002130 may promote osimertinib resistance. Furthermore, in osimertinib-resistant NSCLC cells, has_circ_0002130 induced cell proliferation, survival, and glycolysis through sponging miR-498 to upregulate miR-498 targeted genes GLUT1, HK2, and LDHA (34). Thus, interfering with circRNA expression could be a promising solution to the problem of EGFR-TKIs resistance.

### Resistance to crizotinib

As the first-generation ALK inhibitor, crizotinib showed superior efficacy compared to platinum–pemetrexed chemotherapy in NSCLC with ALK, MET and ROS1 alterations (120). However, acquired crizotinib resistance is a major challenge in NSCLC management. Emerging evidence has manifested that circRNAs are involved in crizotinib resistance. One recent study reported that F-circEA1, a fused circular RNA derived from an EML4-ALK1, promoted tumor proliferation, migration, invasion, and cell cycle progression, as well as crizotinib resistance in NSCLC cells. Besides, knockdown of F-circEA1 significantly inhibited EML4-ALK1 expression and the downstream signaling pathway of ALK (18).

### Resistance to immunotherapy

Antibody-directed therapies against immunological checkpoints, also known as immunological checkpoint inhibitors (ICI), have remarkable effects in the treatment of advanced lung cancer (113). Notably, the blockade of PD-1/PD-L1 has been identified to be effective in NSCLC (121, 122). However, T cell activation and antigen recognition disorders promote resistance to PD-L1 therapy (123). Several studies have illustrated the aberrant expressions of circRNAs have a very close connection with immunotherapy resistance. Chen et al. demonstrated that exosomal circUSP7 contributed to anti-PD1 immunotherapy in NSCLC cells by inhibiting CD8+ T cell function. Mechanistically, circUSP7 increases the expression of SHP2 through sponging miR-934 (11). Another study showed that circFGFR1 was frequently upregulated in NSCLC and ectopic expression of circFGFR1 induced cell proliferation, survival, invasion and immune evasion. More importantly, circFGFR1 increased CXCR4 expression by functioning as a sponge of miR-381-3p, leading to NSCLC resistance to anti-PD-1 therapy (124). Zhang et al. found that circHMGB2 promoted the proliferation of NSCLC and remodeled the TME, limiting the efficacy of PD-1 blockade in NSCLC treatment *via* modulating the miR-181a-5p/CARM1 axis (125). Besides, Ge et al. demonstrated that circ_CELF1 was elevated in primary NSCLC tissues. Circ_CELF1 was able to increase the expression of target gene EGFR through acting as a sponge of miR-491-5p, resulting in NSCLC progression and resistance to immunotherapy (126). These findings indicate that the dysregulation of circRNAs serves as a crucial part of ICI resistance in NSCLC. (The current literatures describing circRNAs in drug resistance and underlying mechanisms are listed in **Table 2**).

**Table 2:** circRNAs function as miRNAs sponge in drug resistance of lung cancer.

Drug	CircRNAs	MiRNAs	Target genes/proteins	Effects	References
Cisplatin	circ_SOX13	miR-3194	MAPRE1	contributes resistance	(39)
	circ_CUL2	microRNA-888-5p	RB1CC1	contributes resistance	(19)
	circ_0002360	miR-6751-3p	ZNF300	contributes resistance	(127)
	hsa_circ_0017639	miR-1296-5p	SIX1	contributes resistance	(107)
	circ_PIP5K1A	miR-493-5p	ROCK1	contributes resistance	(128)
	circ_0058357	miR-361-3p	ABCC1	contributes resistance	(129)
	circ_PRMT5	miR-138-5p	MYH9	enhance sensitivity	(14)
	circ_100565	miR-337-3p	ADAM28	contributes resistance	(16)
	circ_0020123	miR-140-3p	HOXB5	contributes resistance	(130)
	circAKT3	miR-516b-5p	STAT3	contributes resistance	(60)
	circ_ PRMT5	miR‐4458	REV3L	contributes resistance	(40)
	circ_0000079	–	FXR1/PRCKI	contributes resistance	(65)
	hsa_circRNA_103809	miR-377-3p	GOT1	contributes resistance	(105)
	circ_0072083	miR-545-3p	CBLL1	contributes resistance	(131)
	circ_CPA4	let-7 miRNA	PD-L1	contributes resistance	(132)
	circ- CDR1as	miR-641	HOXA9	contributes resistance	(133)
	circ_0076305	miR-296-5p	STAT3	contributes resistance	(92)
Gefitinib	circ_0014235	miR-146b-5p	YAP/PD-L1	contributes resistance	(116)
	circ_MACF1	miR-942-5p	TGFBR2	contributes resistance	(12)
	circ_0001658	miR-409-3p	TWIST1	contributes resistance	(33)
	circ_SETD3	miR-873-5p	APPBP2	contributes resistance	(117)
	hsa_circ_0004015	miR-1183	PDPK1	contributes resistance	(41)
	circRACGAP1	miR-144-5p	CDKL1	contributes resistance	(115)
	circ_102481	miR-30a-5p	ROR1	contributes resistance	(134)
Taxol	hsa_circ_0011298	miR-486-3p	CRABP2	contributes resistance	(135)
	circ_0002360	miR-585-3p	GPRIN1	contributes resistance	(136)
	hsa_circ_0030998	miR-558	MMP1/MMP17	contributes enhance sensitivity	(137)
	hsa_circ_0002483	miR-182-5p	GRB2, FOXO1, and FOXO3	enhance sensitivity	(98)
Docetaxel	circ_0003998	miR-136-5p	CORO1C	enhance sensitivity	(103)
Paclitaxel	circ_0001821	miR-526b-5p	GRK5	contributes resistance	(138)
	circ_0011292	miR-379-5p	TRIM65	contributes resistance	(95)
	circ_ZFR	miR-195-5p	KPNA4	contributes resistance	(96)
	hsa_circ_0002874	miR1273f	MDM2/P53	contributes resistance	(97)
Osimertinib	hsa_circ_0002130	miR-498	GLUT1, HK2, and LDHA	contributes resistance	(34)
	hsa_circ_0005576	miR-512-5p	IGF1R	contributes resistance	(119)
Anti-PD-1	circ-FGFR1	miR-381-3p	CXCR4	contributes resistance	(124)
	circUSP7	miR-934	SHP2	contributes resistance	(11)
	circHMGB2	miR-181a-5p	CARM1	contributes resistance	(139)

## CircRNAs as therapeutic targets to overcome drug resistance in NSCLC

Based on the findings described above, circRNAs have great potential to serve as therapeutic targets to surmount chemo-, TKI- and ICI-resistance in NSCLC. Expression plasmids and RNA interference-based strategies are typical methods for gain-of-function and loss-of-function of circRNAs, respectively. Many scholars conducted plenty of researches on recovering the sensitivity of NSCLC cells to drug therapies through the intervention of circRNAs expression. Wang et al. reported that enforced expression of cirPTK2 reduced CDDP resistance in A549/CDDP and H1299/CDDP cells through regulation of the miR-942/TRIM16 axis (64). Zhang et al. demonstrated that knockdown of circ_0072088 with small hairpin RNA (shRNA) significantly suppressed CDDP resistance in NSCLC cells. As a sponge of miR-944, depletion of circ0072088 led to downregulation of LASP1 (140). Using both small interfering RNA (siRNA) and shRNA, silencing circ0004015 (si-circ0004015 and sh-circ0004015) resulted in inhibiting CDDP resistance in CDDP-resistant NSCLC cells (141). Even more strikingly, some studies showed that targeting circRNA could completely reverse drug resistance by activating the apoptotic pathway. Wang et al. found that ectopic expression of circASK1 attenuated gefitinib resistance *via* its encoded protein ASK1-272a.a, which competes with ASK1 for binding to AKT to suppress AKT-mediated ASK1-Ser83 phosphorylation. As a result, gefitinib sensitivity was restored by activation of the ASK1/JNK/p38 pro-apoptotic signaling in LUAD cells (142). (The circRNAs as potential therapeutic targets for overcoming drug resistance in NSCLC are shown in **Table 3**).

**Table 3:** CircRNAs as potential therapeutic targets for overriding drug resistance in NSCLC.

Intervention	CircRNAs	Pathway	Effects	References
Knockdown of circRNA	circ_0072088	miR-944/LASP1 axis	contributes to CDDP sensitivity in NSCLC	(140)
	Circ_PVT1	miR−429/FOXK1 signaling axis	enhances the sensitivity to cisplatin in NSCLC	(143)
	Circ_PRMT5	miR‐4458/REV3L axis	contributes to CDDP‐ sensitivity	(40)
	circ_0001821	miR-526b-5p/GRK5 axis	suppresses paclitaxel resistance of NSCLC	(138)
enforced expression of circRNAs	circ_PTK2	miR-942/TRIM16 axis	reduced CDDP resistance in NSCLC	(64)
Silencing of circRNAs	circ_0004015	miR198/KLF8 axis	represses CDDP resistance in NSCLC	(141)
	hsa_circ_0074027	miR-379-5p/IGF1 axis	enhances chemosensitivity to docetaxel in NSCLC	(144)
	circ_0014130	miR-545-3p-YAP1 axis	suppresses drug resistance to docetaxol in NSCLC	(102)
	Circ_WHSC1	miR-296-3p/AKT axis	increases NSCLC cell apoptosis	(145)
	Circ_0017639	miR-1296-5p/SIX1	reduces the resistance and CDDP-resistant NSCLC cells	(107)
circRNA activates apoptosis	circ_ASK1	ASK1/JNK/p38 axis	ameliorates gefitinib resistance in NSCLC	(142)

## Discussions and perspectives

In this review, we summarized recent findings and research breakthroughs on the role of specific circRNAs in drug resistance in NSCLC. The study of how circRNAs mediate drug resistance is one of research hot topics (39). It has been documented that circRNAs control cellular processes through several mechanisms which include modulating transcription (23), serving as sponge for miRNAs (134), acting as a platform or sponge for proteins (65), regulating splicing at the same locus (22), forming functional circRNA-protein complexes (65), directly binding to mRNAs to regulate their expression, outcompeting linear mRNAs for protein binding and encoding peptides (5, 146). However, a vast majority of reports in drug resistance-associated circRNAs have focused on their function as miRNA sponge (107, 119). Further investigations are needed to elucidate involvement of other molecular mechanisms of circRNAs in NSCLC drug resistance. In addition, previous studies have concentrated on differential expression and functions of circRANs between drug-resistant and -sensitive NSCLC tumor cells. The role of circRNAs in the TME of drug resistant NSCLC and the effect of circRNAs derived from TME on NSCLC drug resistance remain elusive. Moreover, the mechanisms by which circRNAs are up- or down-regulated in drug resistant NSCLC are also largely unknown. The study of these aspects will further enhance our understanding of the mechanisms of drug resistance and identify potential therapeutic targets to overcome the resistance in NSCLC.

Based on the results from various reports, a number of circRNAs were dysregulated in a single drug resistant NSCLC cell line or patient (97, 147, 148). Future investigations are required to address whether these circRNAs contribute to the drug resistance individually or together, and whether they form networks and which circRNA is a key player within the net to control the resistance. In addition, the strategy to target circRNA is inadequate. Currently, circRNAs are typically silenced using miRNA-based approach (141) and overexpressed using expression vectors (64). However, miRNA molecules have a number of limitations even though nanoparticles or exosomes delivery systems could improve their stability, intracellular entry, and immunogenicity. Moreover, circRNA expression viral plasmids could cause unanticipated side effects. Thus, it is crucial to develop new effective approaches to target circRNAs for overcoming drug resistance in NSCLC.

All in all, based on further extensive studies on the mechanisms of circRNA-mediated drug resistance, modulating circRNAs will be a novel therapeutic approach to conquer NSCLC drug resistance in the future.

## Author contributions

Conceptualization: SJ and JW. Writing, original draft preparation: TY. Writing, review and editing: SJ, QL, FL and XT. Visualization: QS and TY. Supervision: SJ and JW. Funding acquisition: SJ. All authors have read and agreed to the published version of the manuscript.

## Funding

This work was supported by National Natural Science Foundation of China (grant no. 81873249 and 82074360), National Natural Science Foundation of Shandong Province (grant no. ZR2019MH058) and the Young Taishan Scholars Program of Shandong Province (grant no. tsqn201909200).

## Conflict of interest

The authors declare that the research was conducted in the absence of any commercial or financial relationships that could be construed as a potential conflict of interest.

## Publisher’s note

All claims expressed in this article are solely those of the authors and do not necessarily represent those of their affiliated organizations, or those of the publisher, the editors and the reviewers. Any product that may be evaluated in this article, or claim that may be made by its manufacturer, is not guaranteed or endorsed by the publisher.
